# The levonorgestrel intrauterine system versus endometrial ablation for heavy menstrual bleeding: a cost‐effectiveness analysis

**DOI:** 10.1111/1471-0528.16836

**Published:** 2021-07-27

**Authors:** MJ van den Brink, P Beelen, MC Herman, PM Geomini, JH Dekker, KM Vermeulen, MY Bongers, MY Berger

**Affiliations:** ^1^ Department of General Practice and Elderly Care Medicine University of Groningen University Medical Centre Groningen Groningen The Netherlands; ^2^ Department of General Practice University of Maastricht Maastricht University Medical Centre Maastricht The Netherlands; ^3^ Department of Obstetrics and Gynaecology Máxima Medical Centre Veldhoven The Netherlands; ^4^ Department of Epidemiology University of Groningen University Medical Centre Groningen Groningen The Netherlands; ^5^ Department of Obstetrics and Gynaecology Grow Research School for Oncology and Developmental Biology University of Maastricht Maastricht The Netherlands

**Keywords:** Cost‐effective analysis, economic evaluation, excessive uterine bleeding, intrauterine device, menorrhagia, menstruation, mirena, noninferiority trial, novasure

## Abstract

**Objective:**

To evaluate the costs and non‐inferiority of a strategy starting with the levonorgestrel intrauterine system (LNG‐IUS) compared with endometrial ablation (EA) in the treatment of heavy menstrual bleeding (HMB).

**Design:**

Cost‐effectiveness analysis from a societal perspective alongside a multicentre randomised non‐inferiority trial.

**Setting:**

General practices and gynaecology departments in the Netherlands.

**Population:**

In all, 270 women with HMB, aged ≥34 years old, without intracavitary pathology or wish for a future child.

**Methods:**

Randomisation to a strategy starting with the LNG‐IUS (*n* = 132) or EA (*n* = 138). The incremental cost‐effectiveness ratio was estimated.

**Main outcome measures:**

Direct medical costs and (in)direct non‐medical costs were calculated. The primary outcome was menstrual blood loss after 24 months, measured with the mean Pictorial Blood Assessment Chart (PBAC)‐score (non‐inferiority margin 25 points). A secondary outcome was successful blood loss reduction (PBAC‐score ≤75 points).

**Results:**

Total costs per patient were €2,285 in the LNG‐IUS strategy and €3,465 in the EA strategy (difference: €1,180). At 24 months, mean PBAC‐scores were 64.8 in the LNG‐IUS group (*n* = 115) and 14.2 in the EA group (*n* = 132); difference 50.5 points (95% CI 4.3–96.7). In the LNG‐IUS group, 87% of women had a PBAC‐score ≤75 points versus 94% in the EA group (relative risk [RR] 0.93, 95% CI 0.85–1.01). The ICER was €23 (95% CI €5–111) per PBAC‐point.

**Conclusions:**

A strategy starting with the LNG‐IUS was cheaper than starting with EA, but non‐inferiority could not be demonstrated. The LNG‐IUS is reversible and less invasive and can be a cost‐effective treatment option, depending on the success rate women are willing to accept.

**Tweetable abstract:**

Treatment of heavy menstrual bleeding starting with LNG‐IUS is cheaper but slightly less effective than endometrial ablation.

## Tweetable abstract

Treatment of heavy menstrual bleeding starting with LNG‐IUS is cheaper but slightly less effective than endometrial ablation.

## Introduction

Heavy menstrual bleeding (HMB) is a common problem, affecting more than a quarter of women of reproductive age.^1^ HMB not only has a significant impact on a woman’s quality of life, it also puts a heavy economic burden on society.^2^, ^3^ HMB diagnosis is associated with significant direct medical costs and indirect work loss costs.^4^, ^5^

Three decades ago, 60% of women with HMB who were referred to a gynaecologist had a hysterectomy as treatment.^6^ Currently, two less invasive treatments are used and have been shown to reduce the number of hysterectomies performed for HMB:^7^, ^8^ the levonorgestrel intrauterine system (LNG‐IUS), a device that reduces menstrual blood loss by local release of progestogen in the uterine cavity and that can be inserted in primary or secondary care, and endometrial ablation (EA), a technique performed by the gynaecologist, which reduces menstrual blood loss by destroying the uterine endometrium.

Compared with oral medical treatment, the LNG‐IUS shows a greater improvement of quality of life^9^ and also seems cost‐effective^10^, ^11^, ^12^ depending on the quality of life questionnaire used.^13^ Second‐generation (non‐hysteroscopic) endometrial techniques seem to be more cost‐effective than first generation (hysteroscopic) techniques. They require less operating time, can be used more often with local anaesthesia, and have fewer complications.^14^, ^15^ The few data available suggest that the LNG‐IUS is potentially cheaper and more effective than first‐generation ablation techniques.^16^ The LNG‐IUS seems dominant to the second‐generation EA techniques: microwave and thermal balloon ablation in terms of costs and quality of life.^11^, ^17^, ^18^, ^19^ However, discontinuation rates of the LNG‐IUS are high (36–60%)^9^, ^20^ and definitive evidence on the effectiveness of the LNG‐IUS compared with the most used second generation EA (bipolar radiofrequency) is lacking.^21^ Because the LNG‐IUS procedure is reversible and less invasive compared with EA and easily feasible in general practice, we investigated whether a strategy starting with the LNG‐IUS (Mirena, Bayer HealthCare Pharmaceuticals, Berlin, Germany) was non‐inferior and cost‐effective compared with a strategy starting with bipolar radiofrequency EA (NovaSure, Hologic, Marlborough, MA, USA) in the treatment of HMB after a 24‐month follow‐up.

## Methods

### Study design

A cost‐effectiveness analysis from a societal perspective with a 24‐month time horizon was performed alongside a multicentre randomised controlled non‐inferiority trial (MIRA trial). Women with HMB, aged 34 or older, were randomised to either a strategy starting with the LNG‐IUS or a strategy starting with EA after written informed consent. Participants were allowed to undergo a re‐intervention. Women were excluded if they had a wish for pregnancy, abnormal cervix cytology in the past 5 years, intracavitary fibroids, polyps or large intramural fibroids. Women were recruited at participating general practices (*n* = 197) or outpatient gynaecology departments (*n* = 26) in the Netherlands. The study was approved by the ethics committee of the Academic Medical Centre Amsterdam, the Netherlands (registration number 2011_372), and by the board of directors of each of the participating hospitals. A more detailed description of the design of the MIRA trial has been published elsewhere.^22^

### Treatment procedures

The 52‐mg levonorgestrel IUS (Mirena) was inserted by either a general practitioner in his/her practice or by a gynaecologist at the outpatient department without anesthesia.^23^ EA was performed with a bipolar radiofrequency device (NovaSure^®^), a second‐generation ablation technique.^24^ The procedure was performed by a gynaecologist in the operation room under general or spinal anaesthesia or at the outpatient department under local anaesthetic or conscious sedation.

### Assessment of effect

A core outcome set for HMB has not yet been developed.^25^ Our primary outcome was mean blood loss at 24 months after randomisation, measured with the Pictorial Blood Assessment Chart (PBAC)‐score.^26^ The non‐inferiority margin was set at 25 points. Because the distribution of the PBAC‐scores at 24 months’ follow‐up was found to be highly skewed with a large proportion of zero‐scores, confidence intervals around the estimated mean difference and the *P*‐value for non‐inferiority were calculated using bootstrapping (10 000 replications). To test the robustness of our result, we added a secondary analysis with a zero‐inflated negative‐binomial (ZINB) model.^27^ In this cost‐effectiveness analysis we present the mean PBAC‐scores with the corresponding standard deviations because they provide information on the variability in PBAC‐scores. The parameters of the ZINB model do not directly represent the difference between two means and are therefore hard to interpret for clinicians and policymakers. These results are not directly useful in a cost‐effectiveness analysis and are presented elsewhere.^27^Secondary outcomes included the proportion of women with successful blood loss reduction (PBAC‐score ≤75 points), patient satisfaction (measured with a 5‐point Likert scale) and quality of life, measured with the disease‐specific Menorrhagia Multi‐Attribute Scale (MMAS) and the generic Short Form 36 (SF‐36) Health Survey Questionnaire.^28^, ^29^, ^30^, ^31^, ^32^ A preference‐based measure of health (SF‐6D) was derived from the SF‐36 questionnaire.^33^, ^34^ More details on all secondary outcomes have been published elsewhere.^22^, ^27^

### Assessment of costs

Both direct medical and direct‐ and indirect non‐medical costs were included in the analyses. Relevant direct cost components were costs of the LNG‐IUS and EA treatment, medication, re‐interventions, GP and specialist consultations, hospital admission, diagnostic tests and home care, consisting of both professional care as well as informal care. Data on healthcare use and re‐interventions were collected from patient questionnaires and medical records. Costs of the treatment procedures LNG‐IUS and EA were calculated according to the bottom‐up principle following the Dutch guidelines for cost studies and using the Dutch tariff in the year of analysis (2018).^35^ Indirect non‐medical costs such as sick leave and loss of productivity at work were measured with the Short Form‐Health and Labour Questionnaire (SF‐HLQ) at baseline, 3, 6, 12 and 24 months of follow‐up.

### Handling of missing data

Missing data on the primary and secondary effect outcomes were not imputed. For missing sick leave data, no productivity loss was assumed if the following data on treatment effect were present at that moment of follow‐up: amenorrhoea (PBAC‐score = 0) or the patient was satisfied with the treatment effect (secondary outcome) or menstrual blood loss was not influencing daily activities (MMAS‐score = 100, maximum score for disease‐specific quality of life).^31^

### Cost‐effectiveness analysis

Menstrual blood loss (mean PBAC‐score after 24 months) as well as the average costs per patient over a 24‐month time horizon were measured for each treatment strategy to calculate the incremental cost‐effectiveness ratio (ICER). The ICER expressed the incremental costs per one point difference in PBAC‐score (LNG‐IUS compared with EA). Bootstrap analyses (5000 replications) were performed to create alternative 95% confidence intervals surrounding the point estimate of the ICER, and to create cost‐effectiveness planes.

### Cost–utility analysis

We planned to perform a cost–utility analysis expressing the incremental costs per Quality Adjusted Life Year (QALY) based on the SF‐6D score, derived from the SF‐36 questionnaire. However, the proportion of patients that completed the SF‐36 questionnaire at all follow‐up time points (baseline, 3, 6, 12 and 24 months) was 44.7% in the LNG‐IUS group and 62.3% in the EA group. The proportion of missing data was too large (>20%) to perform multiple imputation and therefore reliable utilities could not be calculated and a meaningful cost–utility analysis could not be performed.

### Secondary analyses

We performed two secondary analyses: (1) a cost‐effectivity analysis with the proportion of women with a PBAC‐score ≤75 points as a measure of effect and (2) a cost‐effectivity analysis with the proportion of women that were satisfied as a measure of effect.

### Sensitivity analyses

We performed the following sensitivity analyses to explore the robustness of the results: (1) calculation of costs under the assumption that all LNG‐IUS insertions were performed in primary care and (2) calculation of costs under the assumption that all EAs were performed at the outpatient department under local anaesthesia (paracervical block).

### Patient and public involvement

The funding source (ZonMw) has involved stakeholders, including patients as the target group, in the assessment of the relevance of our study. We have incorporated their suggestions into our study protocol. Furthermore, patients were actively involved in the development of the patient information letter. Patients were not involved in other stages of the development of the research.

## Results

### Participants and treatment procedures

In all, 270 women were randomised to a treatment strategy starting with the LNG‐IUS (*n* = 132) or EA (*n* = 138) (Figure S1). In the LNG‐IUS group, 122 women successfully received the allocated intervention. In the EA group, 130 women started the allocated intervention, but in seven women the intervention did not succeed because of problems during the procedure. Reasons for not receiving the allocated treatment are presented in the flow diagram in Figure S1. Baseline characteristics of the participating women are presented in Table 1.

**Table 1:** Baseline characteristics

	Endometrial ablation (*n* = 138)	LNG‐IUS (*n* = 132)
Age, mean (SD)	45.3 (4.9)	44.7 (4.6)
BMI, mean (SD)	27.8 (5.8)	27.5 (5.4)
Number of vaginal deliveries, mean (SD)	1.7 (1.0)	1.8 (1.1)
Caesarean section	23 (17%)	30 (23%)
Previous uterus surgery	16 (11.6%)	11 (8.3%)
Duration of HMB in months, median (IQR)	12.0 (5.0–24.0)	12.0 (6.0–28.0)
Previous treatment		
Non‐hormonal*	30 (21.7%)	26 (19.7%)
Hormonal**	52 (37.7%)	54 (40.9%)
Anticoagulants	2 (1.4%)	5 (3.8%)
Presence of dysmenorrhea	87 (67%)	89 (73%)
Duration of menstruation in days, median (IQR)	7.0 (6.0–10.0)	8.0 (6.0–10.5)
PBAC‐score, mean (SD)	630.0 (551.8)	616.3 (524.3)

BMI, body mass index; HMB, heavy menstrual bleeding; LNG‐IUS, levonorgestrel intrauterine system; PBAC, Pictorial Blood Assessment Chart.

Data are number of women (%), unless otherwise indicated.

* Tranexamic acid or nonsteroidal anti‐inflammatory drugs.

** Oral contraceptives, progestogens, LNG‐IUS, GnRH agonist or NuvaRing^®^.

LNG‐IUS insertion was performed nine times in general practice (7.4%). In the remaining cases, LNG‐IUS insertion was performed by the gynaecologist at the outpatient department (89.3%) or the operating room (3.3%). The majority of LNG‐IUS were inserted without anaesthesia, in four women the procedure was performed under general anaesthesia (Table S1).

EA was performed by the gynaecologist in the operating room in 58% of cases and at the outpatient department in 42% of cases. Almost half of the patients received general anaesthesia, 38% of women a paracervical block, and 13% spinal anaesthesia. Twenty per cent of the women in the EA group (*n* = 27) received a re‐intervention compared with 35% of the women in the LNG‐IUS group (*n* = 44). Additionally, 11 women discontinued the LNG‐IUS without a re‐intervention (discontinuation rate: 43%). Hysterectomy rates were comparable between the two strategies (7.1% LNG‐IUS versus 10.1% EA; relative risk [RR] 0.70, 95% CI 0.31–1.56) (Table S1).

### Treatment effect

A complete case analysis was performed with all participants for whom the primary outcome (PBAC‐score at 24 months) was available: 115/132 (87%) in the LNG‐IUS group and 132/138 (96%) in the EA group. The mean PBAC‐score at 24 months was 64.8 in the LNG‐IUS group versus 14.2 in the EA group (difference 50.5, 95% CI 4.3–96.7). Non‐inferiority of the LNG‐IUS (based on a pre‐set margin of 25 points) could not be demonstrated (non‐inferiority *P* = 0.87). Menstrual blood loss decreased to a PBAC‐score ≤75 points in 100/115 women (87%) in the LNG‐IUS group and in 124/132 women (94%) in the EA group (RR 0.93, 95% CI 0.85–1.01). In the LNG‐IUS group, 74/100 women (74%) were satisfied with the treatment effect compared with 98/116 women (84%) in the EA group after 24 months (RR 0.88, 95% CI 0.76–1.01). Longitudinal data analysis of disease‐specific quality of life did not show a significant difference between the two treatment strategies (mean difference MMAS summary score 3.3; 95% CI −0.5 to 7.1).

### Costs

The mean costs per patient after 24 months of the strategy starting with EA was €3,465 and for the strategy starting with the LNG‐IUS was €2,285. The largest cost item was the primary intervention, followed by re‐interventions and work absenteeism (Table 2).

**Table 2:** Mean costs per treatment group

	Costs EA (*n* = 132) Mean in € (SD)	Costs LNG‐IUS (*n* = 115) Mean in € (SD)	Cost difference Mean in € (95% CI*)
Intervention	2,352.03 (492.46)	602.00 (362.89)	−1,750 (−1,880 to −1715)
GP consultations	14.61 (39)	16.21 (40)	2 (−14 to 18)
Specialist consultations	38.75 (113.20)	60.65 (168.92)	22 (−21 to 27)
Hospital admission	129.13 (379.92)	143.99 (587.60)	15 (−94 to 30)
Intensive Care admission	18.39 (211.23)	0.00 (0.00)	18 (0 to 0)
Absenteeism	519.66 (1733.33)	495.35 (2180.93)	−24 (−266 to 179)
Professional home care	2.79 (32.05)	2.67 (28.62)	0 (0 to 11)
Other paid home care	30.82 (315.67)	41.79 (419.94)	11 (−91 to 2)
Medication	5.00 (28.09)	14.78 (94.85)	10 (3 to 5)
Other re‐interventions	354.01 (985.10)	907.25 (1524.50)	553 (24 to 535)
Total costs to 24 months	3,465.19 (2,887.61)	2,284.70 (3,738.70)	−1,180 (−2,097 to −1,111)

EA, endometrial ablation; GP, general practitioner; LNG‐IUS, levonorgestrel intrauterine system.

Data are presented in means (SD or 95% confidence interval).

* Confidence interval determined by bootstrapping.

### Cost–effectiveness analysis

#### Primary analysis

**Figure 1:**
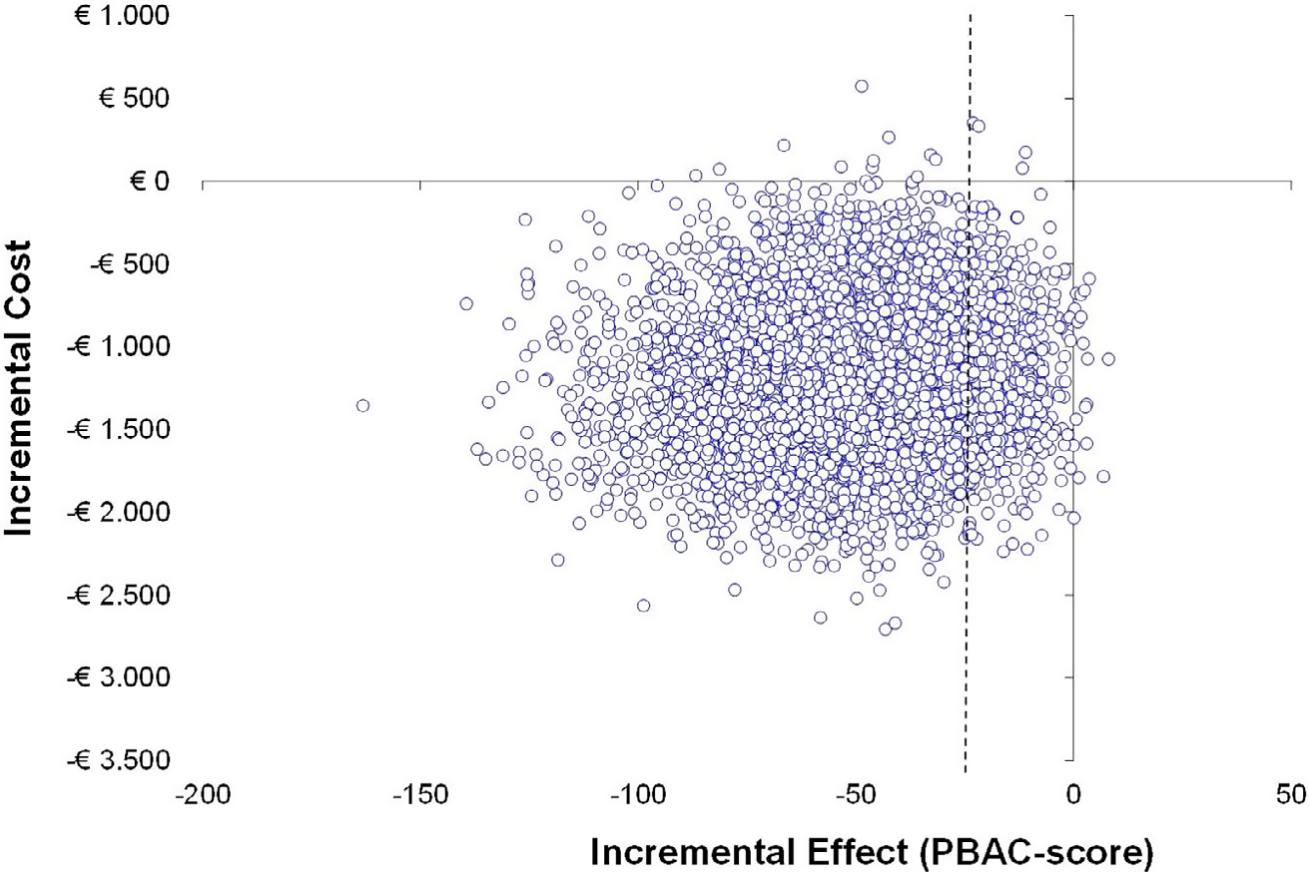
Cost‐effectiveness plane. PBAC, Pictorial Blood Assessment Chart. A negative difference in PBAC‐score represents a higher PBAC‐score (less effect) at 24 months in the levonorgestrel intrauterine system (LNG‐IUS) group than with endometrial ablation (reference treatment strategy). Vertical dotted line represents the chosen non‐inferiority margin of 25 PBAC‐points. Incremental costs presented in Euros, incremental effect presented in PBAC‐points after 24 months.

The strategy starting with the LNG‐IUS was less effective (mean difference: 50.5 PBAC‐points) and less costly (−€1,180; 95% CI −€2,097 to −€1,111) compared with the strategy starting with EA after 24 months. EA costs €23 per additional PBAC‐point reduction of menstrual blood loss (ICER: €23; 95% CI €5–111) (Table 3, Figure 1).

**Table 3:** Effects and costs of LNG‐IUS and endometrial ablation

	Endometrial ablation (*n* = 132) Mean (SD)	LNG‐IUS (*n* = 115) Mean (SD)	Difference Mean (95% CI*)
Effect (PBAC‐score at 24 months)	14.2 (43.4)	64.8 (251.0)	50.5 (4.3–96.7)
Costs (24 months)	€3,465 (2888)	€2,285 (3739)	−€1,180 (−€2,097 to −€1,111)
ICER			€23 (€5 to €111)

ICER, incremental cost‐effectiveness ratio; LNG‐IUS, levonorgestrel intrauterine system; PBAC, Pictorial Blood Assessment Chart.

Data are presented in means (SD or 95% confidence interval).

* Confidence interval determined by bootstrapping.

Figure 1 shows that 14% of the bootstrap replications falls in the quadrant within the non‐inferiority margin and below the *X*‐axis, indicating a 14% chance that the LNG‐IUS has lower costs with comparable effectiveness compared with EA.

#### Secondary analyses

##### Secondary analysis 1: PBAC‐score ≤75 points

The success rate (PBAC‐score ≤75 points) of the LNG‐IUS strategy was 7% lower compared with the success rate of the EA strategy. Our primary analysis showed a cost difference of €1,180 between the two strategies. EA costs €169 per additional 1% of women who are successfully treated (Table S2).

##### Secondary analysis 2: patient satisfaction

Patient satisfaction at 24 months was 10% lower in the LNG‐IUS strategy compared with the EA strategy, with a cost difference of €932 in favour of the LNG‐IUS. There is a 92.5% probability that the LNG‐IUS is less costly with lower patient satisfaction and 4.4% probability that the LNG‐IUS is less costly with a higher patient satisfaction (Figure S2). The EA strategy costs €89 (95% CI −€335 to €607) per additional 1% of women who are satisfied with the treatment result (Table S3).

### Sensitivity analyses

#### Sensitivity analysis 1: all LNG‐IUS insertions in primary care

In the LNG‐IUS strategy, the mean costs of the primary intervention were reduced to €347 (compared with €602 in the primary analysis, see Table 2) and the total costs were reduced to €2,030. We assumed that other cost items and the effect data would remain the same. The ICER increased to €28 for the EA strategy per additional PBAC‐point reduction compared with the LNG‐IUS strategy (Table S4).

#### Sensitivity analysis 2: EA at the outpatient department with local anaesthesia

The mean costs of the primary intervention in the EA strategy were reduced to €2,241 (compared with €2,352 in the primary analysis, see Table 2) and the mean costs of re‐interventions in the LNG‐IUS strategy were reduced to €873 (compared with €907 in the primary analysis, see Table 2). The ICER decreased to €22 for the EA strategy per additional PBAC‐point reduction compared with the LNG‐IUS strategy (Table S5).

## Discussion

### Main findings

In this cost‐effectiveness analysis from a societal perspective, a strategy starting with the LNG‐IUS was cheaper (‐€1,180) but resulted in less reduction of blood loss (mean difference PBAC‐score at 24 months: 50.5 points) compared with a strategy starting with second‐generation EA (Novasure®) over a 24‐month time horizon. The incremental cost‐effectiveness ratio (ICER) for EA compared with LNG‐IUS was €23 per additional PBAC‐point blood loss reduction. This ICER increased to €28 under the assumption that all LNG‐IUS insertions were performed in primary care, and decreased to €22 under the assumption that all EAs were performed at the outpatient department with local anaesthesia. Expressed as the percentage of women with successful treatment (PBAC ≤75), the ICER was €169 per 1% additional success rate for EA compared with LNG‐IUS. Expressed as the percentage of women satisfied with the treatment strategy, the ICER was €89 per additional 1% satisfaction for EA compared with the LNG‐IUS.

### Strengths and limitations

Strengths of our study are the long follow‐up of 2 years and the bottom‐up calculation of direct medical and indirect costs from clinical trial data, which means calculating each individual cost item. This gives a more accurate estimate of the total costs of a strategy than a top‐down calculation. We believe our study provides a realistic estimate of the number of re‐interventions after LNG‐IUS and EA in the long‐term. Previous studies had a shorter follow‐up of 1 year^16^ or have used theoretic modelling (e.g. Markov model).^11^, ^18^, ^19^, ^36^ Such models are based on several theoretical assumptions which might affect their results. Although HMB treatment by general practitioners and gynaecologists in the Netherlands is comparable to other countries, one must realise that the healthcare system with associated costs in the Netherlands can differ from costs in other countries.^37^

To test the robustness of our results, we performed a sensitivity analysis under the assumption that all LNG‐IUS insertions were performed in primary care, and a sensitivity analysis under the assumption that all EAs were performed at the outpatient department under local anaesthesia. Accommodation costs were included in a fixed overhead percentage of 44%, according to the Cost Guide for Economic Evaluations in Healthcare.^35^ Ideally, we would like to differentiate this overhead percentage for the type of setting where the procedure is performed, but data on these differences are lacking. The actual cost savings when performing procedures in primary care or at the outpatient department versus the operating room can therefore be larger than our sensitivity analyses show.

### Interpretation

To our knowledge, this is the first study comparing the LNG‐IUS with bipolar radiofrequency EA in terms of cost‐effectiveness. Evidence so far is based on the comparison of the LNG‐IUS with other second‐generation ablation techniques (microwave, thermal balloon ablation). Our study showed that a treatment strategy starting with the LNG‐IUS is cheaper than a strategy starting with EA after 2 years of follow‐up, even if taking into consideration the 43% discontinuation rate for LNG‐IUS. This result is consistent with previous studies comparing the LNG‐IUS with other second‐generation ablation techniques.^11^, ^17^, ^18^, ^19^ Most of those studies used quality of life or patient satisfaction as the primary measure for effectiveness instead of menstrual blood loss reduction. There is no consensus on the most appropriate outcome measure to use when assessing the cost‐effectiveness of HMB treatment.^38^ We have chosen the PBAC‐score as our primary outcome because it is an objective measure with a high predictive value for both satisfaction and the chance for re‐intervention.^39^, ^40^ In previous studies, the LNG‐IUS dominated EA because satisfaction rates were similar and/or most QALYs were gained in the LNG‐IUS group. The satisfaction rates and longitudinal data analysis of disease‐specific quality of life (MMAS‐scores) in our trial showed no significant differences between the two treatment strategies. When looking at patient satisfaction (secondary analysis), we could not conclude that the LNG‐IUS dominated the EA strategy because the bootstrapped 95% confidence interval of the cost difference included zero.

## Conclusions

### Clinical implications

In our study, the mean PBAC‐score at 24 months was 50 points higher (95% CI 4.3–96.7) in the LNG‐IUS group than in the EA group. We could not demonstrate non‐inferiority nor inferiority of a strategy starting with the LNG‐IUS compared with a strategy with EA because the 95% confidence interval exceeded the predetermined non‐inferiority margin of 25 points. This makes it difficult to interpret when the LNG‐IUS strategy would be cost‐effective. The cost‐effectiveness plane indicates that there is a 14% chance that the strategy starting with the LNG‐IUS is non‐inferior and less costly compared with a strategy starting with EA. However, there is a substantial chance that the strategy with the LNG‐IUS, although cheaper, results in a (clinical relevant) higher PBAC‐score after 24 months compared with a strategy with EA. The National Institute for Health and Clinical Excellence (NICE) in the UK generally accepts a cost of £20,000 to £30,000 (€22,660–33,990) per additional QALY gained. We calculated the costs per 1 point difference in PBAC‐score, but the price society is willing to pay for an additional decrease in PBAC‐score has not been established.

The majority of women in our trial had successful treatment after 24 months (94% versus 87% in the EA and LNG‐IUS group, respectively). If an 87% chance of success is found to be acceptable, starting with the LNG‐IUS may be a cost‐effective treatment strategy. It is important to discuss the chance of successful blood loss reduction with women who are offered both treatment options, together with other outcomes such as comparable satisfaction rates and quality of life scores. The results of our study are based on a trial in which most LNG‐IUS were inserted in secondary care. In the Netherlands, almost all general practitioners are competent to insert intrauterine devices. Although LNG‐IUS insertions for contraceptive purposes are increasing in general practice, the number of LNG‐IUS insertions for HMB is still low.^41^ In the Nordic countries, the UK and France, IUS use has gradually increased,^42^ whereas in other countries, the IUS appears to be underused, in part due to a lack of primary care providers trained in device insertion.^43^, ^44^ As our sensitivity analysis indicates that an increase in LNG‐IUS insertions in primary care will lead to lower costs for society, IUS treatment in primary care should be encouraged more. In secondary care, EA is internationally increasingly performed at the outpatient department under local anaesthesia, reducing the cost of the ablation strategy.

### Research implications

We performed a cost‐effectiveness analysis with a time horizon of 2 years. As the LNG‐IUS has to be replaced after 5 years and some of the randomised women might be postmenopausal after 5 years of follow‐up, it would be interesting to perform a cost‐effectiveness analysis with a follow‐up of 5 years. However, it has been shown that most (surgical) re‐interventions due to treatment failure occur within 2 years of the initial treatment.^45^

In conclusion, a treatment strategy starting with the LNG‐IUS is less costly for society than a strategy starting with EA in women with HMB. Although the amount of blood loss reduction after 24 months is less compared with EA, and the total re‐intervention rate is higher, both strategies lead to successful menstrual blood loss reduction after 24 months in the majority of women (94% and 87%, respectively) and high patient satisfaction. Depending on the success rate women are willing to accept, starting with the LNG‐IUS can be a cost‐effective treatment. Compared with EA, the LNG‐IUS is a reversible and less invasive treatment option for HMB, with a contraceptive effect. It is important to counsel women about the different characteristics of the two interventions and about the expected treatment results in terms of menstrual blood loss reduction, satisfaction, improvement of daily life activities and risk of a (surgical) re‐intervention.

### Disclosure of interests

MJ van den Brink and P Beelen report a grant from ZonMw, the Netherlands Organisation for Health Research and Development, to conduct the study. MY Bongers reports financial relationships with Gynesonics (device reduction in clinical trials), outside the submitted work. MY Bongers is a member of the communication team of Bayer and the advisory board of Hologic. The remaining authors report no conflicts.

### Contribution to authorship

MvdB, MH, PG, JD, MBo and MBe were involved in the conception and design of the study. MvdB, MH and PB were involved in data collection for the study. MvdB, PB and KV performed the statistical analyses. MvdB and PB wrote the first draft of the report. All authors participated in manuscript drafting and approved the final draft.

### Details of ethics approval

The MIRA trial was approved by the ethics committee of the Academic Medical Centre Amsterdam, the Netherlands (date: 8 March 2012; registration number 2011‐372).

### Funding

ZonMw, the Netherlands Organisation for Health Research and Development, grant number 171202001.

## Supporting information


**Figure S1**. Flow diagram.

**Figure S2**. Cost‐effectiveness plane patient satisfaction.

**Table S1**. Intervention—endometrial ablation and LNG‐IUS.
**Table S2**. Effects and costs LNG‐IUS and endometrial ablation—secondary analysis.
**Table S3**. Effects and costs LNG‐IUS and endometrial ablation—secondary analysis.
**Table S4**. Effects and costs LNG‐IUS and endometrial ablation—sensitivity analysis.
**Table S5**. Effects and costs LNG‐IUS and endometrial ablation—sensitivity analysis.

Supplementary Material

Supplementary Material

Supplementary Material

Supplementary Material

Supplementary Material

Supplementary Material

Supplementary Material

Supplementary Material

## Data Availability

The data that support the findings of this study are available from the corresponding author upon reasonable request.
