# Supplementary figures and images for: The levonorgestrel intrauterine system versus endometrial ablation for heavy menstrual bleeding: a cost‐effectiveness analysis
Source: BJOG. 2021 Jul 27;128(12):2003–11. doi: 10.1111/1471-0528.16836 (PMC8518490; doi:10.1111/1471-0528.16836)

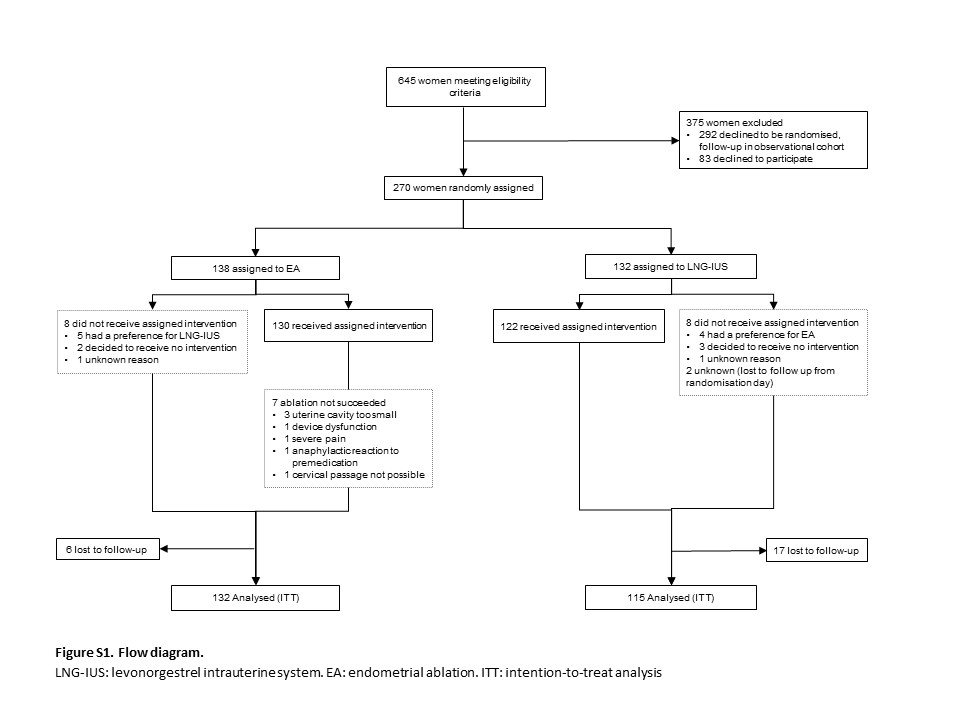

Supplement: Supplementary file 1 — Figure S1. Flow diagram. [file BJO-128-2003-s004.jpg]

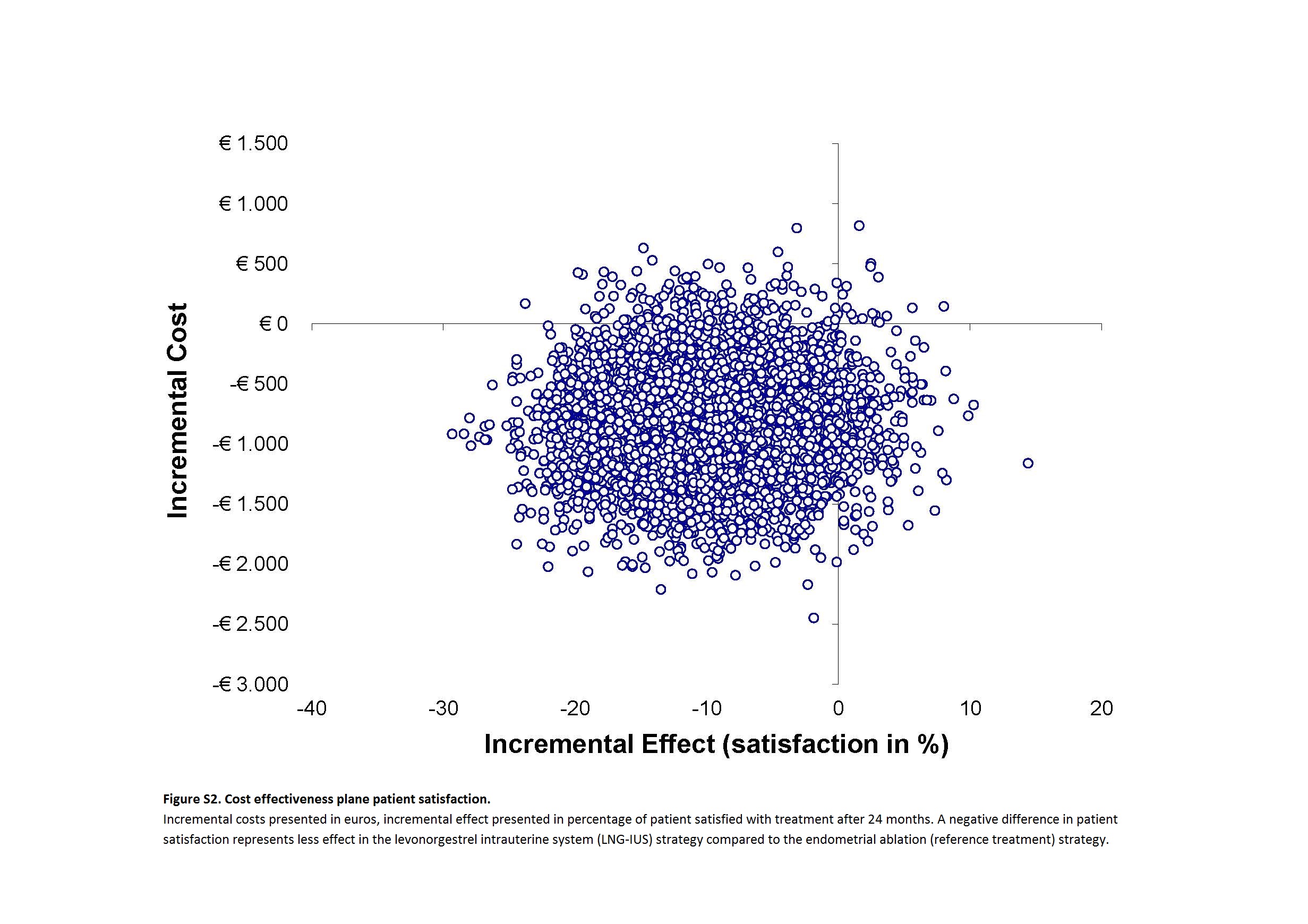

Supplement: Supplementary file 2 — Figure S2. Cost‐effectiveness plane patient satisfaction. [file BJO-128-2003-s008.jpg]
