# Supplementary material for: The levonorgestrel intrauterine system versus endometrial ablation for heavy menstrual bleeding: a cost‐effectiveness analysis
Source: BJOG. 2021 Jul 27;128(12):2003–11. doi: 10.1111/1471-0528.16836 (PMC8518490; doi:10.1111/1471-0528.16836)
Supplement: Supplementary file 3 — Table S1. Intervention—endometrial ablation and LNG‐IUS. Table S2. Effects and costs LNG‐IUS and endometrial ablation—secondary analysis. Table S3. Effects and costs LNG‐IUS and endometrial ablation—secondary analysis. Table S4. Effects and costs LNG‐IUS and endometrial ablation—sensitivity analysis. Table S5. Effects and costs LNG‐IUS and endometrial ablation—sensitivity analysis. [file BJO-128-2003-s007.docx]

**Supporting Information files**

## **Table S1. Intervention - Endometrial ablation and LNG-IUS.**

|  | **Endometrial ablation**  **(n=138)** | | **LNG-IUS**  **(n=132)** | |  |
| --- | --- | --- | --- | --- | --- |
| Procedure performed | 130 | (94%) | 122 | (92%) | |
| Performed by*: |  |  |  |  | |
| Gynaecologist | 130 | (100%) | 113 | (93%) | |
| Outpatient department | 54 | (42%) | 109 | (89%) | |
| Operating room | 76 | (58%) | 4 | (3.3%) | |
| General practitioner | n/a |  | 9 | (7.4%) | |
| Anaesthesia* |  |  |  |  | |
| Spinal | 17 | (13%) | 0 | (0%) | |
| General | 63 | (48%) | 4 | (3.3%) | |
| Paracervical block | 49 | (38%) | 0 | (0%) | |
| Not applicable | 1 | (0.8%) | 118 | (97%) | |
| Number of women with re-intervention** | 27 | (20%) | 44 | (35%) | |
| Total number of re-interventions** | 34 | (24.6%) | 67 | (52.8%) | |
| Endometrial ablation | 1 | (0.7%) | 31 | (24.4%) | |
| First generation | 1 | (0.7%) | 1 | (0.8%) | |
| Second-generation | 0 | (0%) | 30 | (23.6%) | |
| Hysterectomy | 14 | (10.1%) | 9 | (7.1%) | |
| LNG-IUS | 3 | (2.2%) | 1 | (0.8%) | |
| Drug treatment** | 15 | (10.9%) | 24 | (18.9%) | |
| Hysteroscopic surgery | 1 | (0.7%) | 0 | (0%) | |
| Myomectomy | 0 | (0%) | 2 | (1.6%) | |

Values are given as n (%). LNG-IUS: levonorgestrel intrauterine system. * Percentages are calculated over number of performed procedures: Endometrial ablation group N=130, LNG-IUS group N=122. ** Women could receive ≥ one re-intervention in the course of 24 months

**Table S2. Effects and costs LNG-IUS and endometrial ablation – secondary analysis 1.**

|  | **Endometrial ablation** (n=132)  (mean; SD) | **LNG-IUS** (n=115)  (mean; SD) | **Difference**  (mean; 95% CI) |
| --- | --- | --- | --- |
| Effect (PBAC-score ≤ 75 in %) | 94% | 87% | -7% (-22% – 8%) |
| Costs | €3,465 (2888) | €2,285 (3739) | -€1,180 (-€2,097 – -€1,111)* |
| ICER |  |  | €169 (-€392 – €1,174)* |

LNG-IUS: levonorgestrel intrauterine system. PBAC: Pictorial Blood Assessment Chart. ICER: Incremental Cost-Effectiveness Ratio. Data are presented in means (SD or 95% confidence interval) * Confidence interval determined by bootstrapping.

**Table S3. Effects and costs LNG-IUS and endometrial ablation – secondary analysis 2.**

|  | **Endometrial ablation** (n=116)  (mean; SD) | **LNG-IUS** (n=100)  (mean; SD) | **Difference**  (mean; 95% CI) |
| --- | --- | --- | --- |
| Effect (satisfaction in %) | 85% | 75% | -10% (-21% – 1%) |
| Costs | €3,330 (2954) | €2,398 (3963) | -€932 (-€2,894 – €848)* |
| ICER |  |  | €89 (-€335 – €607)* |

LNG-IUS: levonorgestrel intrauterine system. ICER: Incremental Cost-Effectiveness Ratio. Data are presented in means (SD or 95% confidence interval) * Confidence interval determined by bootstrapping.

**Table S4. Effects and costs LNG-IUS and endometrial ablation – sensitivity analysis 1.**

|  | **Endometrial ablation** (n=132)  (mean; SD) | **LNG-IUS** (n=115)  (mean; SD) | **Difference**  (mean; 95% CI*) |
| --- | --- | --- | --- |
| Effect  (PBAC-score at 24 months) | 14.2 (43.4) | 64.8 (251.0) | 50.5 (4.3 – 96.7) |
| Costs | €3,465 (2888) | €2,030 (3747) | -€1,435 (-€2,317 – -€1,334) |
| ICER |  |  | €28 (€9 – € 133) |

LNG-IUS: levonorgestrel intrauterine system. PBAC: Pictorial Blood Assessment Chart. ICER: Incremental Cost-Effectiveness Ratio. Costs are calculated under the assumption that all LNG-IUS are inserted in primary care. Data are presented in means (SD or 95% confidence interval) * Confidence interval determined by bootstrapping.

**Table S5. Effects and costs LNG-IUS and endometrial ablation – sensitivity analysis 2.**

|  | **Endometrial ablation** (n=132)  (mean; SD) | **LNG-IUS** (n=115)  (mean; SD) | **Difference**  (mean; 95% CI*) |
| --- | --- | --- | --- |
| Effect  (PBAC-score at 24 months) | 14.2 (43.4) | 64.8 (251.0) | 50.5 (4.3 – 96.7) |
| Costs | €3,354 (2903) | €2,247 (3710) | -€1,107 (-€2,008 – -€998) |
| ICER |  |  | €22 (€5 – €108) |

LNG-IUS: levonorgestrel intrauterine system. PBAC: Pictorial Blood Assessment Chart. ICER: Incremental Cost-Effectiveness Ratio. Costs are calculated under the assumption that all endometrial ablations are performed at the outpatient department with local anaesthesia. Data are presented in means (SD or 95% confidence interval) * Confidence interval determined by bootstrapping.
